# Clinical and Histopathological Characteristics of Prostate Cancer Patients Taken to Palliative Transurethral Prostate Resection

**DOI:** 10.7759/cureus.5740

**Published:** 2019-09-24

**Authors:** Juan P Rojas-Manrique, Angie Ramírez Ramírez, Luis Miguel Becerra Méndez, Jose G Ramos Ulloa, Carlos Riveros, Rodolfo Varela Ramirez

**Affiliations:** 1 Urologic Oncology, Instituto Nacional de Cancerologia, Bogotá, COL

**Keywords:** prostate cancer, transurethral resection of the prostate, prostate-specific antigen(psa)

## Abstract

Introduction

In prostate cancer (PCa) patients who have been treated with radiotherapy and/or androgen deprivation therapy (ADT), palliative transurethral resection of the prostate (TURP) is a management option in the presence of lower urinary tract symptoms (LUTS). The present work seeks to describe the clinical and histopathological characteristics of patients with PCa taken to palliative TURP.

Methods

An observational, descriptive and retrospective study of patients with PCa who underwent palliative TURP for the relief of obstructive urinary symptoms at an oncology reference center between January 2006 and June 2014 was performed. Among the included patients were those with localized PCa treated with radiotherapy and those with advanced PCa with or without metastasis who had previously received ADT.

Results

Sixty-six patients with a diagnosis of PCa taken to palliative RTUP were identified. Fifty patients (78.4%) were received some type of ADT, seven patients (10.7%) received curative radiotherapy along with adjuvant ADT, five patients (7.8%) were previously treated with only radiotherapy, and two patients (3.1 %) had received no prior management and thus were taken to bilateral orchiectomy along with palliative TURP in a single surgical act. With regard to the pathology reports, tumor tissue was found in 50 patients (76%), and no tumor was observed in the remaining 16 patients (24%). In one case (1.5%), the Gleason score (GS) could not be determined due to the effects of orchiectomy. Under-staging in the grade group was evidenced in 23 patients (46.9%), over-staging in three patients (6.3%), and no difference in 23 patients (46.9%), when compared to the initial GS at biopsy. The mortality rate and the incidence of TURP syndrome were low (3.1% and 1.5%, respectively). A 46% reduction in the mean serum prostate-specific antigen (PSA) value was documented when the preoperative and postoperative values were compared.

Conclusion

A decrease in the serum PSA levels was observed after palliative TURP, and despite having received ADT, it was possible to determine tumor pathology in the resected tissue, being able to identify a greater grade group compared the GS at the time of diagnosis. The palliative TURP proved to be a safe procedure to relieve LUTS in patients with advanced PCa, with a low morbidity and mortality rate.

## Introduction

In patients with prostate cancer (PCa), lower urinary tract symptoms (LUTS) may occur during any stage of the disease and some patients may even experience urinary retention [1]. In PCa patients who have been treated with radiotherapy and/or androgen deprivation therapy (ADT), palliative transurethral resection of the prostate (TURP) is a management option in the presence of LUTS [2]. As medical therapy has a limited role in the treatment of LUTS in patients with PCa, surgery continues to be the first line of management [3]. Furthermore, TURP remains the treatment of choice for symptomatic benign prostatic hyperplasia (BPH) because of its low rate of long-term complications even after 10 years of follow-up [4]. The first reports concerning its use in malignancy date back to 1991 when Mazur and Thompson reviewed 41 patients undergoing “channel” TURP for outlet obstructive symptoms in the context of advanced PCa. They reported a 22% reintervention rate and a 5% incidence of stress incontinence [5]. The present work sought to describe the characteristics of patients with PCa and LUTS taken to palliative TURP at an oncology reference center in Bogota, Colombia.

## Materials and methods

An observational, descriptive, and retrospective study was carried out in which patients with PCa who were taken to palliative TURP for relief of LUTS between January 2006 and June 2014 were included. Additionally, the included patients diagnosed with PCa with the localized disease had been treated with radiotherapy, while those with advanced disease (with or without metastases) had received or were receiving some type of ADT (pharmacological or surgical).

Within the clinical characteristics of the study group, the preoperative and postoperative serum PSA levels were evaluated, along with the clinical stage according to the TNM classification, Gleason scores (GS), grade groups, and the type of treatment received prior to the palliative TURP. The histopathology of the initial diagnostic biopsy and that of the resected tissue after the palliative TURP were also evaluated.

The analysis was carried out with frequency measures for each of the variables and the functional clinical outcomes were classified as a new episode of urinary retention, urethral stricture and/or urinary incontinence, as well as the mortality rate associated with the surgical procedure and the incidence of TURP syndrome.

## Results

Between 2006 and 2014, a total of 66 patients were identified. The mean age was 66 ( 52 to 84 ) years. The mean serum PSA level at the time of palliative TURP was 17.77 (0.005 to 100) ng/ml. The most common indication for the palliative TURP was the presence of obstructive urinary symptoms (54.5%). Likewise, most had previously received symptomatic treatment via the placement of a urethral catheter (42.4%). With regards to the pre-established PCa, most patients were staged as either T2 or T3 (54.6%), with a grade group of 1 (31.8%). Fifty-five patients (83.3%) were receiving ADT due to advanced disease, while 11 patients (16.7%) had received radiotherapy for localized disease.

The preoperative clinical characteristics of the 66 patients are shown in Table 1. The mean follow-up was 10 months with a range between four and 24 months.

**Table 1:** Clinical characteristics of patients TURP, transurethral resection of the prostate; GnRH, gonadotropin-releasing hormone; PSA, prostate-specific antigen; N/A, not available; ng/mL, nanograms per milliliter

	Frequency (%)
Patients	66
Mean age (range)	66 (52-84)
Indication for TURP	
Obstructive urinary symptoms	36 (54.5%)
Acute urinary retention	24 (36.3%)
Cystolithiasis	3 (4.5%)
Hematuria	3 (4.5%)
Previous management	
Urethral catheter	28 (42.4%)
Alpha blocker	28 (42.4%)
None	10 (15.2%)
Treatment prior to TURP	
Orchiectomy	43 (65.1%)
GnRH analog	12 (18.1%)
Radiotherapy	11 (16.7%)
T stage	
T1	14 (21.2%)
T2	18 (27.3%)
T3	18 (27.3%)
T4	16 (24.2%)
Gleason Grade Group	
1	21 (31.8%)
2	8 (12.1%)
3	8 (12.1%)
4	13 (19.7%)
5	15 (22.7%)
N/A	1 (1.6%)
Mean serum PSA prior to surgery (ng/ml) [range]	17.77 (0.005 – 100)

The clinical results obtained after the palliative TURP are presented in Table 2. The mean volume of the resected tissues was 16.5 (3-60) cc. A reduction of 46% in the mean serum PSA levels was documented between the preoperative value (17.77 ng/ml) and the postoperative value (8.33 ng/ml). The mortality rate associated with the surgical procedure and the incidence of TURP syndrome were low (3.03% and 1.52%, respectively).

**Table 2:** TURP characteristics TURP, transurethral resection of the prostate; PSA, prostate-specific antigen; ng/mL, nanograms per milliliter

	Frequency (%)
Mean resected volume in cc (range)	16.5 (3-60)
Postoperative mortality	
Yes	2 (3.0%)
No	64 (96.9%)
TURP syndrome	
Yes	1 (1.5%)
No	65 (98.5%)
Postoperative mean serum PSA (ng/ml)	8.33 (0.003-87)
Complications	
Urinary retention	1 (1.52%)
Urethral stricture	4 (6.06%)
Urinary incontinence	2 (3.06%)

In the histopathological analysis, tumor tissue was found in 50 patients (76%), whereas no tumor was evidenced in 16 patients (24%). Among the cases with evidence of malignancy, in one patient (1.5%) the GS could not be determined due to the changes observed after orchiectomy. It was noted that the majority of patients treated with radiotherapy had a benign pathology report, while the majority of patients treated with ADT (pharmacological or surgical) showed evidence of adenocarcinoma (Table 3).

**Table 3:** Correlation between treatment type for prostate cancer and pathology results GnRH, gonadotropin-releasing hormone

	Orchiectomy	Radiotherapy	GnRH analog
Adenocarcinoma	34 (51.5%)	5 (7.5%)	10 (15.5 %)
Adenocarcinoma with changes due to hormone therapy	1 (1.5%)	0	0
Hiperplasia	8 (12.0%)	6 (9.0%)	2 (3.0%)

Among those who had histopathological staging, the majority of the patients had a GS of 5 in the initial biopsy (15 patients, 30.6%), as well as in the postoperative grade group (24 patients, 44.8%). There were no patients with postoperative grade group of 1. In addition to the 49 cases that could be classified, under-staging in the grade group was evidenced in 23 patients (46.9%), over-staging in three patients (6.3%), and no difference in 23 patients (46.9%), when compared to the initial GS at biopsy (Table 4).

**Table 4:** Comparison between the preoperative and postoperative Gleason grade groups GG, grade groups

		Postoperative grade group			
		GG2	GG3	GG4	GG5
Gleason score at biopsy	GG1	1 (2.1%)	3 (6%)	1 (2.1%)	4 (8.1%)
	GG2	1 (2.1%)	2 (4.1%)	3 (6%)	1 (2.1%)
	GG3	0 (0%)	3 (6%)	2 (4.1%)	2 (4.1%)
	GG4	1 (2.1%)	0 (0%)	6 (12.4%)	4 (8.1%)
	GG5	0 (0%)	1 (2.1%)	1 (2.1%)	13 (26.4%)

## Discussion

Lower urinary tract obstruction is possible in patients with PCa. While most patients are initially treated with hormone therapy, it can take too long for the therapy to exert the desired effect [1]. In a study of 35 patients with advanced PCa and bladder outlet obstruction symptoms treated with orchiectomy, it was documented that 68% of patients finally managed to achieve spontaneous urination, but 46% required catheterization 21 to 60 days after orchiectomy [2].

Transurethral resection of the prostate continues to be the treatment of choice for the relief of LUTS due to prostatic disease [4]. In patients with PCa, the cause of LUTS is not always clear and tumor growth may lead to both storage and voiding symptoms, this being a form of disease progression. As there is no established protocol for the management LUTS in patients with PCa, surgery remains the first line of treatment [3].

The results of conducting “channel” TURP were initially published in the study by Mazur and Thompson in 1991 [5]. In its analysis of 41 patients, 46% received radiotherapy as part of the treatment while the rest were treated with hormonal therapy. The immediate results after TURP were favorable, all patients achieved spontaneous urination without perioperative mortality, but 22% of patients required surgical reintervention and two patients died within six months of follow-up [5]. In another study of 24 patients taken to palliative TURP, 58% were treated with radiotherapy while the rest received hormonal management [1]. After the surgical procedure, the urinary symptoms improved according to the International Prostate Symptoms Score (IPSS) from 21 to 11, and the mean urinary flow rate went improved from 7.3 cc/sec to 9.6 cc/sec [1]. Twenty-nine percent of the patients required a second TURP and 21% required prolonged urethral catheterization [1]. In our study, urinary retention occurred in 1.52% of cases after palliative TURP, requiring prolonged urinary catheterization. In addition, the incidence of postoperative urethral stricture and stress urinary incontinence was 6.06% and 3.06%, respectively.

Patients with a positive histopathology report for malignancy in the resected tissue during TURP have significantly lower survival rates compared to those with negative reports, independent of the resected volume [6]. Analyses have shown that these patients have more advanced tumors at the time of diagnosis and this worsens the prognosis by itself, not by the surgical procedure [6]. Some authors suggest that palliative TURP disseminates PCa mainly in stages T3 and T4, and hematogenous dissemination of the tumor has been documented [6]. This observation has not yet been confirmed and it cannot be assured that the progression of the disease increases. In our study, although no tumor progression or survival was evaluated, a high percentage (75.76%) of tumor pathology was found in the resected tissue and even with a greater grade group than the preoperative GS in most of the cases, which can indicate the progression of the disease despite the pre-established management.

Although patients were receiving ADT as part of their treatment for advanced PCa, a high percentage of malignancy was found in the tissue resected during TURP, which would indicate a greater tumor involvement compared to the clinical stage. Likewise, in a minority of patients, it was not possible to assess the GS due to the changes generated after ADT.

In a reviewed case series, the postoperative Gleason value of the patients who underwent TURP was found in 17 (89.4%) patients after 21 procedures [1]. Positive pathology for adenocarcinoma was found in 11 procedures (13 patients); one of moderate grade (GS: 5-7) and the rest of high grade (GS: 8-10) [1]. In another study, malignancy was found in the histopathological report of 16 out of 18 palliative TURP specimens (88%); low (GS: 2-4), a moderate and high grade in one, eight and seven procedures, respectively [7]. These findings can be compared with ours, since prostate adenocarcinoma was documented in 68.2% of the resected pathology specimens, along with an increase in the presence of high-grade GS, from 45.5% preoperative to 82.7% postoperative. This would indicate the progression of the disease with the passage of time despite being treated with ADT, with a Gleason under-staging of 46.9%. Likewise, in the patients in whom no tumor pathology was found after palliative TURP, the majority of patients had a low-risk preoperative GS.

The perioperative mortality rate evidenced in this study was 3.03%, comparable to the results of other series (2.2%) of palliative TURP [6]. Likewise, the incidence of TURP syndrome was low in our study (1.52%), similar to the large series for BPH ranging from 1 to 3% [8]. Although TURP is a safe procedure in patients with PCa, there is evidence that even in patients with locally advanced disease, this procedure poses more risk compared to patients with conventional TURP due to benign disease [9-10]. In addition, despite being a therapeutic option to improve bladder outlet obstruction, it has no effect on ureteral obstruction. [9-10]. In relation to prognosis, two studies concluded that the fact of requiring a palliative TURP is an independent adverse prognostic factor, thus proposing a more active and careful treatment [11-12]. Conversely, a recently published study of metastatic PCa patients found that performing palliative TURP in addition to administering ADT, could significantly improve cancer-specific survival (CSS) compared with patients who only received ADT, when PSA ≥65 ng/ml, GS ≥8, and bone metastases ≤5 [13]. Although palliative TURP is the treatment of choice in patients with LUTS and PCa, only ADT can relief retention and decrease post voiding residual urine volume over a period of time, and can obviate the need for channel TURP in some patients [14].

In our study, the postoperative serum PSA decreased with respect to the preoperative value, going from an average value of 17.77 ng/ml to 8.33 ng/ml, a finding that has not been documented in similar studies. Most articles only report the preoperative serum PSA and the serum PSA at the time of initial diagnosis. A 46% decrease in the post-surgical serum PSA values ​​was evidenced in our study, noting that patients continued adherence to the same ADT management they had preoperatively.

We consider the size of our study group one of the strengths of this study, the highest among the reviewed published studies for this article. Nevertheless, prospective studies are recommended, with larger sample sizes, longer follow-up times, in addition to evaluation of the overall and CSS rates for this population.

## Conclusions

In this study, palliative TURP was found to be a safe procedure to relieve LUTS in patients with advanced PCa. Low morbidity and mortality rates and low rates of postoperative urinary retention, urethral stricture, and urinary incontinence were recorded. A decrease in the postoperative serum PSA levels was observed. In spite of having received ADT, it was possible to identify tumor pathology in the resected tissue, being able to quantify the value of Gleason score and finding greater high-grade tumor pathology, regardless of the type of ADT that the patient had previously received.

## References

[1] Crain DS, Amling CL, Kane CJ (2004). Palliative transurethral prostate resection for bladder outlet obstruction in patients with locally advanced prostate cancer. J Urol.

[2] Fleischmann JD, Catalona WJ (1985). Endocrine therapy for bladder outlet obstruction from carcinoma of the prostate. J Urol.

[3] Gnanapragasam VJ, Kumar V, Langton D, Pickard RS, Leung HY (2006). Outcome of transurethral prostatectomy for the palliative management of lower urinary tract symptoms in men with prostate cancer. Int J Urol.

[4] Varkarakis J, Bartsch G, Horninger W (2004). Long-term morbidity and mortality of transurethral prostatectomy: a 10-year follow-up. Prostate.

[5] Mazur AW, Thompson IM (1991). Efficacy and morbidity of "channel" TURP. Urology.

[6] Marszalek M, Ponholzer A, Rauchenwald M, Madersbacher S (2007). Palliative transurethral resection of the prostate: functional outcome and impact on survival. BJU Int.

[7] Chang CC, Kuo JY, Chen KK (2006). Transurethral prostatic resection for acute urinary retention in patients with prostate cancer. J Chin Med Assoc.

[8] Madersbacher S, Marberger M (1999). Is transurethral resection of the prostate still justified?. BJU Int.

[9] Piper C, Epplen R, Erps TV, Pfister DJKP, Porres D, Heidenreich A (2012). Palliative transurethral resection in men with castration-resistant prostate cancer (CRPC): minimally invasive procedure with minimal morbidity?. J Clin Oncol.

[10] Pelletier J, Cyr SJ, Julien AS, Fradet Y, Lacombe L, Toren P (2018). Contemporary outcomes of palliative transurethral resection of the prostate in patients with locally advanced prostate cancer. Urol Oncol.

[11] Krupski TL, Stukenborg GJ, Moon K, Theodorescu D (2010). The relationship of palliative transurethral resection of the prostate with disease progression in patients with prostate cancer. BJU Int.

[12] Choi SY, Ryu J, You D, Jeong IG, Hong JH, Ahn H, Kim CS (2018). Oncological effect of palliative transurethral resection of the prostate in patients with advanced prostate cancer: a propensity score matching study. J Cancer Res Clin Oncol.

[13] Qu M, Zhu F, Chen H (2019). Palliative transurethral resection of the prostate in patients with metastatic prostate cancer: a prospective study of 188 patients. J Endourol.

[14] Sood R, Singh RK, Goel H, Manasa T, Khattar N, Tripathi MC (2017). Can androgen-deprivation therapy obviate the need of channel transurethral resection of the prostate in advanced prostate cancer with urinary retention? A prospective study. Arab J Urol.

